# Development and Implementation of National Real-Time Surveillance System for Suicide Attempts in Uruguay

**DOI:** 10.3390/ijerph22030420

**Published:** 2025-03-13

**Authors:** Karina Rando, Laura de Álava, Denisse Dogmanas, Matías Rodríguez, Matías Irarrázaval, Jose Luis Satdjian, Alejandra Moreira

**Affiliations:** 1 Ministerial Office, Ministry of Public Health, Montevideo 11200, Uruguay; jsatdjian@msp.gub.uy; 2 National Mental Health Department, Ministry of Public Health, Montevideo 11200, Uruguay; ldealava@msp.gub.uy (L.d.Á.); ddogmanas@msp.gub.uy (D.D.); matiasrodriguez@msp.gub.uy (M.R.); amoreira@msp.gub.uy (A.M.); 3 Pan American Health Organization (PAHO/WHO), Washington, DC 20037, USA; irarrazmat@paho.org

**Keywords:** surveillance system, real time data, self-harm, suicide attempt, suicide prevention

## Abstract

Suicide is a major global public health concern and one of the leading causes of death worldwide. Previous suicide attempts are one of the strongest predictors of future suicide risk, underscoring the need for effective prevention strategies. Central to these strategies is the establishment of robust surveillance systems that deliver accurate and timely data at both national and local levels. This article describes the development, implementation, and progress of Uruguay’s national real-time surveillance system for suicide attempts, which comprehensively covers all emergency departments across the country. The Ministry of Public Health conducts continuous monitoring of suicide attempt cases using a real-time surveillance system. This article also explores the implications of this system for suicide prevention at various levels and discusses future challenges and opportunities for optimizing its use to enhance public health interventions.

## 1. Introduction

Suicide is one of the leading causes of death worldwide. Uruguay stands out as one of the countries with the highest suicide mortality rate in the Americas. In 2023, the age-standardized mortality rate was 19.4 per 100.000 inhabitants (*n* = 763). The highest rates of suicide (not adjusted for age) are observed in older adults from 75 to 79 and young people aged 20 to 25 [1]. In this context, the prevention and management of suicidal behavior has become a key public health policy priority. Since 2011, the National Honorary Commission for Suicide Prevention, led by the Ministry of Public Health, has developed national strategies for suicide prevention, with guidance from experts representing diverse academic disciplines. One of the major objectives of the National Suicide Prevention Strategy 2021–2025 is to “Enhance the national surveillance system, evaluation processes, and conduct high-quality research pertaining to suicidal behavior” [2] (p. 27).

Previous suicide attempts are a significant risk factor for suicide [3,4]. Updated data on suicide and suicide attempts are essential in underpinning interventions and assessing their effectiveness. Access to real-time information on self-harm and suicide attempts enables the early detection of individuals at risk and the appropriate allocation of resources for their care. This relies on the establishment and maintenance of surveillance systems that provide accurate and timely national and local information on suicide and suicide attempts.

Public health surveillance systems are fundamental for suicide prevention. Systematic surveillance of suicide attempts is particularly crucial, allowing not only for the identification of at-risk populations, but also for evaluating the effectiveness of preventive interventions [5].

In addition, data collected by surveillance systems can be used to develop indicators for national mental health information systems and to show progress towards reaching global targets, such as reducing the suicide rate by one third by 2030, as outlined in the United Nations Sustainable Development Goals [6] and the WHO’S Comprehensive Mental Health Action Plan 2013–2030 [7].

Some countries have established regional or national systems for recording self-harm and suicide attempts [8]. Ireland was the first to develop a National Self-Harm Registry in 2002 [9]. Other countries such as the United States, Canada, Australia, Belgium, Denmark, France, New Zealand, Norway, Sweden, and the United Kingdom have integrated suicide attempt and self-harm data within broader national health surveillance systems [5]. Uruguay has high-quality and promptly available data on suicide mortality, extracted from death certificates and coded by the Department of Vital Statistics of the Ministry of Public Health using the International Classification of Diseases (ICD) codes [8,10].

Uruguay implemented a mandatory national registry for suicide attempts in 2013. Initially conducted in paper format, this registry included 34 variables. Some were completed by the healthcare professional providing first-contact care to the individual who had experienced a suicide attempt at an emergency department, in emergency home care, or in primary care services. Subsequently, additional variables were completed by a mental health professional. Despite its potential, various issues were identified, including underreporting due to difficulties in completing records, logistical challenges, and the lack of a plan for systematic data consolidation. The need to improve the registry system was highlighted [2].

A digital registry system was developed by the Ministry of Public Health in October 2022 that provides real-time access to information on suicide attempts across the country.

The aim of this article is to present the development of a national real-time surveillance system for suicide attempts, the progress in its implementation, and its implications at different levels.

## 2. Materials and Methods

### 2.1. Setting and Coverage

Uruguay is a small country with a population of 3,444,263 in 2023 [11]. The population is composed of 48% men and 52% women. In total, 40% live in Montevideo, the capital city, making it the largest urban agglomeration in the country, while only 3% of the population resides in rural areas [11]. Life expectancy at birth in 2024 is estimated as 75 years for men and 81.5 years for women [12]. Since 2021, the number of births has been lower than the number of deaths, indicating an acceleration in the process of population decline.

Health coverage in Uruguay totals 98.5% [13]. In 2007, the National Integrated Health System (SNIS—for its acronym in Spanish) was implemented, reforming the health system. The SNIS is composed of public and private healthcare providers who offer explicitly guaranteed universal coverage. The guiding principles are universality, accessibility, sustainability, and quality of healthcare. It emphasizes health promotion and prevention, developed within the framework of the Primary Health Care strategy. Leadership and governance of the health system come under the Ministry of Public Health.

As part of the SNIS’s implementation, Uruguay approved a national regulation plan for psychological treatments and psychosocial interventions, which mandates the provision of psychotherapeutic and psychosocial treatments by all healthcare providers within the country. This includes interventions for people who have attempted suicide and their families. There are specific national mandatory guidelines for the management and follow-up of individuals experiencing suicide attempts within the SNIS. Also, the Ministry of Public Health has recently approved a new regulation that mandates a reduction in costs for commonly used antidepressants (Sertraline, Escitalopram, and Fluoxetine).

The development of the national real-time surveillance system for suicide attempts is led by the Ministry of Public Health as part of a comprehensive strategy to prevent suicide. This system is being implemented in all emergency departments across the country.

### 2.2. Definition of Suicide Attempt

The following definition of suicide attempt is used by the system: “any act whose main purpose is to try to end one’s own existence, whether this act is clearly manifested by the person and/or is the result of a health professional diagnosis” [14] (p. 1). This definition includes different kinds of suicide attempt and underlying motives.

It was developed by a task force group of experts and was originally agreed for the registration of suicide attempts that were carried out in paper format.

### 2.3. Inclusion and Exclusion Criteria

Following recommendations by the Pan American Health Organization [PAHO] [5], standardized surveillance inclusion and exclusion criteria for the registry of case presentations were established.

Inclusion criteria:

- All emergency department presentations for suicide attempts;
- Diagnostic confirmation by mental health professional;
- All methods of self-harm with suicidal intent.

Exclusion criteria:

- Self-harm without confirmed suicidal intent;
- Suicidal ideation without attempt;
- Accidental injuries;
- Fatal cases (suicides).

### 2.4. Stages for System Development

An operational group was established within the Ministry of Public Health to define the necessary steps for the development and implementation of the national surveillance system for suicide attempts. This group was coordinated by professionals from the National Mental Health Department, with active participation and commitment of the Minister and Vice Minister of Public Health. Their leadership was crucial for the management and consolidation of the system.

The recommendations from the PAHO manual [5] served as a guide for supporting the establishment and maintenance of a surveillance system for hospital-presenting suicide attempts. A review of data registration systems for suicide and suicide attempts in other countries was conducted.

The previously mentioned mandatory suicide attempt registration in paper format, implemented in 2013, was evaluated to identify its strengths and limitations. This evaluation served as the basis for developing the digital registration system [15].

A technical group of suicide prevention experts, including representatives from different stakeholders (academic institutions, healthcare providers, clinicians), convened to provide insights into decision-making regarding the selection of variables for inclusion in the system and its application. This process involved multiple stages, including group work sessions and the distribution of an online survey requesting the prioritization of the most relevant variables.

Ultimately, specific criteria were established for data collection. The value of information on suicide attempts at various levels was emphasized, particularly for the epidemiological characterization of suicidal behavior and for the identification and monitoring of individuals at risk of suicide. Special attention was given to the time required for healthcare workers to complete the registration and the potential workload this might impose, as these factors can contribute to underreporting. As a result, it was determined that the registration process should be quick, concise, and practical.

### 2.5. Stages for Implementation

The implementation of the national surveillance system for suicide attempts in Uruguay has been carried out through a series of strategic and coordinated steps.

The enactment of Ordinance No. 1323/022 by the Ministry of Public Health mandated its implementation in all healthcare providers nationwide, with suicide attempt registration required within 24 h in the emergency department following the attempt.

To manage the implementation of the system, a working team was established comprising different areas of the Ministry of Public Health, including professionals from the National Mental Health Department, e-government, and the Department of Health Surveillance. This team worked closely with all healthcare providers within the National Health System to coordinate the necessary steps and ensure the proper functioning of the system.

Access to the system is restricted exclusively to healthcare professionals authorized by the Ministry of Public Health to register suicide attempts. All authorized personnel received a short training, which provided instructions on the use of the new digital registration system. These professionals maintain direct communication with the Ministry of Public Health to resolve any technical issues or inquiries efficiently. Additionally, the mental health departments of each healthcare provider have real-time access to the information, allowing them to coordinate appropriate follow-ups for these individuals.

### 2.6. Ethical Considerations and Data Protection

The system operates under stringent data protection and privacy protocols aligned with international standards for health surveillance systems [16].

Each record is anonymized through an automatically generated unique identifier, following the recommendations by the WHO for suicide surveillance data [7]. Access is restricted to authorized healthcare personnel, with all system access being logged and audited.

The system complies with national personal data protection regulations [17]. Data handling procedures follow the guidelines established by WHO [16] for sensitive health data management in surveillance systems.

### 2.7. Quality Control

An essential step in monitoring suicide attempts is the validation of recorded information. To ensure accuracy and consistency in suicide attempt surveillance, a comprehensive quality assurance protocol has been established.

Following the registration of a suicide attempt by any healthcare professional in the emergency department, the initial validation process requires confirmation of a diagnosis by mental health specialists. Records that do not meet the established inclusion criteria are excluded. Additionally, a central team at the Health Surveillance Department of the Ministry of Public Health conducts daily reviews of the records, verifying the completeness and internal consistency of the data.

Ongoing monitoring activities are critical to maintaining data quality over time. These activities include quarterly audits to systematically assess data reliability and continuous feedback provided to mental health services.

Moreover, quality indicators are utilized to assess the performance of the surveillance system. These include achieving a 100% record completeness rate, ensuring registration timeliness within 24 h of the event, and maintaining documented follow-up for all cases

## 3. Results

### 3.1. National Surveillance System for Suicide Attempt Characteristics

The national system provides access to real-time information on suicide attempts. The data collected include demographic information on each patient, the circumstances of their presentation, and the attention received. The main variables included are described in Table 1.

### 3.2. Implementation Outcomes

**Figure 1:**
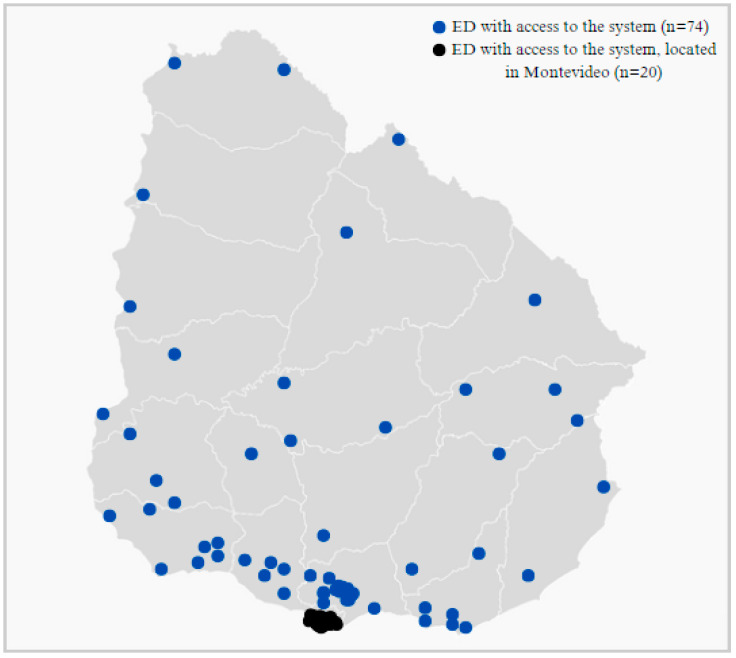
Geographical location of 97 emergency departments with access to the system in Uruguay.

The system covers all (*n* = 97) emergency departments across the country, ensuring comprehensive national implementation (see Figure 1). Twenty of them are in Montevideo. The country’s existing e-government infrastructure was used to facilitate real-time data entry and access.

Authorized healthcare workers (*n* = 1114) can access the system, and mental health departments within each healthcare provider have real-time access to the data, enabling timely intervention and follow-up care for at-risk individuals. The majority of these healthcare workers are nurses or nursing assistants (35.8%), followed by specialty physicians (19.6%), general practitioners (18.6%), and administrative staff (12.6%). The average time required to complete registration is 5 min.

Based on the information provided by the system, the Ministry of Public Health monitors compliance with the established mandatory guidelines for the care and follow-up of each suicide attempt [14,18]. This monitoring is essential for developing timely interventions and understanding the epidemiology of suicide attempts.

The guidelines establish steps for managing suicide attempts at the emergency department level, planning for discharge, ensuring continuity of care, and providing outpatient treatment and community follow-up. It includes a minimum of six months of follow-up interventions. The period immediately following discharge from an inpatient unit is one of high risk for recurrence or suicide, making it crucial to maintain clinical contact [19,20]. In 2024, the guidelines were strengthened with a pay-for-performance action plan for healthcare providers to improve quality and reduce waiting times for individuals who have recently attempted suicide. Specific actions were implemented for discharge planning, including phone call follow-up assessments at 2 days and thirty days post-suicide attempt.

Sanctions on healthcare providers are applied when they do not comply with these guidelines.

**Figure 2:**
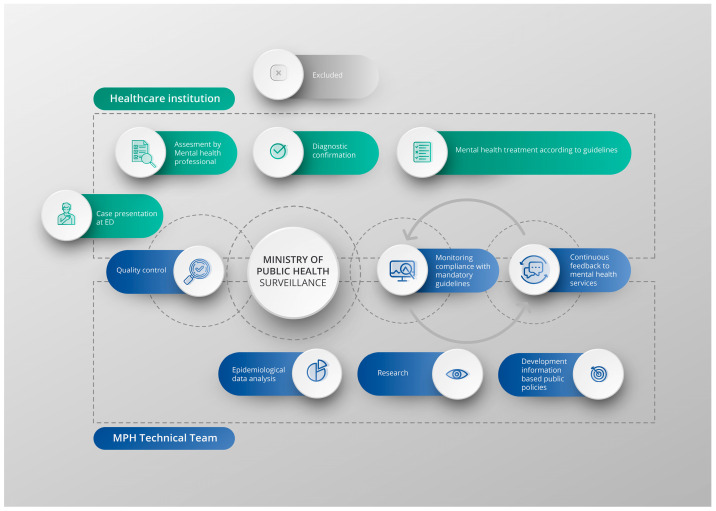
Flowchart of suicide attempt registration, clinical management, and monitoring process.

Figure 2 illustrates the actions taken by the Ministry of Public Health and the healthcare institution following the registration of the suicide attempt.

### 3.3. Limitations

The national surveillance system for suicide attempts does not capture all suicide attempts, as it primarily focuses on those presenting at emergency departments. International evidence suggests that for every suicide, there may be as many as 20 suicide attempts [3]. Although individuals who attempt suicide—or their families—frequently seek help at hospital emergency departments, evidence indicates that a significant number do not, and some may never enter the healthcare system [5,8].

Data from the first year of implementation of the digital registration system in Uruguay reveal a ratio of 6.4 to 1 between suicides and known suicide attempts in 2023, which is significantly lower than this international estimate. The characteristics of this system do not allow for the identification of suicide attempts in other settings, nor do they ensure that individuals at risk receive adequate care.

Another limitation to note is that the options for the method of self-harm do not include all those covered by ICD-10. The most common methods were prioritized to streamline the registry process, but suicide attempt registration systems that utilize the full ICD-10 list allow for more robust international comparisons of the most common methods across different populations.

Undoubtedly, there are additional risk factors and variables relevant to this topic that could provide valuable insights at an epidemiological level [4]. However, as previously mentioned, this system was designed to prioritize a quick, concise, and practical registration process to ensure its feasibility.

## 4. Discussion

This work provides an in-depth analysis of the development and implementation of a national surveillance system for suicide attempts in Uruguay, highlighting its implications at multiple levels. Initially, the system was paper-based and included many variables of interest. However, its implementation was not successful. Transitioning to an effective digital system required political will, infrastructure, and reducing the number of variables to be collected.

Few countries have information on suicide attempts and self-harm at national level.

Ireland, as a pioneer in developing a nation-specific system, served as a valuable model for Uruguay. Further efforts are needed globally to expand national suicide attempt monitoring systems [5,7,21].

In Uruguay, the development and implementation of this specific system adhered to international recommendations for public health surveillance systems [5,22,23]. These guidelines also emphasize the importance of its integration into the broader national health surveillance framework to enhance its impact on public health. The country’s existing e-government infrastructure has been a significant advantage in advancing this goal.

The developed system offers benefits at different levels that should be highlighted. The information allows for continuous monitoring of each person who attempts suicide, and the care received at national mental health services. For the first time, it enables the collection of real-time data (maximum 24 h) on suicide attempts nationwide, and establishes a national baseline. This is a significant step in enhancing data on suicidal behavior and developing information-based public policies.

At an epidemiological level, the system gathers information that allows for a better understanding of the epidemiology of suicide attempts. The system’s data are actively utilized at the macro level by policymakers within the Ministry of Public Health for the design, monitoring, and evaluation of public health interventions. They also enable trend observation, assessment of variations across different time periods, regions, and populations, and the development of targeted strategies. The system provides valuable opportunities for cross-analysis with other information sources, which aligns with the insights of Robinson et al. [24] regarding the implementation of a similar system in a region of Australia.

At a clinical level, mental health workers have quick access to information on suicide attempts registered in emergency departments, facilitating case management and follow-up care.

A relevant step moving forward is the continuous training and capacity building of healthcare workers to accurately identify and register suicide attempts in emergency departments [9]. This will improve the quality of the data obtained. The Ministry of Public Health offers virtual and free courses and clinical guidelines on suicide prevention, detection, and management for first-level care and emergency department workers.

With respect to the number of cases reported compared to international statistics, our primary hypothesis is that this discrepancy is due to underreporting. It has been observed that some hospitals are still not fully documenting all suicide attempts presented in their emergency departments. Nevertheless, it is essential to further examine this hypothesis while also considering alternative explanations, such as variations in healthcare-seeking behavior, social stigma, or other contextual factors that could influence the reporting and recording of suicide attempts.

Factors contributing to suicide and suicide attempts can vary significantly between countries [25]. Conducting national research is essential for generating high-quality scientific knowledge on suicide attempts and their predictors within the context of Uruguay.

It is important to plan a periodic review and continuous improvement of the surveillance system to ensure its quality and sustainability over time [9]. According to the Consolidated Framework for Implementation Research [26], several factors have been identified as critical to the successful implementation of the suicide attempt surveillance system. Key facilitators include external elements such as high-level political support and a favorable regulatory framework, as well as system characteristics like integration with existing infrastructure and a user-friendly interface. Within the implementation process, continuous training and permanent technical support are indispensable in building capacity and maintaining engagement among stakeholders.

Several challenges observed during the implementation of the system in Uruguay mirror those reported in other studies [26], such as organizational resistance to change, the need for technological updates, and the need for reliable connectivity across different regions. Coordinated efforts will be essential in addressing these barriers and fully realizing the potential of this national surveillance system. Furthermore, international collaboration and networking could be beneficial in sharing best practices, providing technical support, and fostering innovation to overcome these challenges effectively.

## 5. Conclusions

This system represents a significant advancement in suicide attempt surveillance in Uruguay and the region. Our initial findings indicate that the model is likely viable and useful, though requires continuous attention to data quality and sustained stakeholder commitment. Future enhancements should prioritize integration with other health information systems and the expansion of analytical capabilities to more effectively support decision-making in suicide prevention. It is hoped that, over the coming years, these efforts will allow for improvement alongside the ongoing actions of the National Suicide Prevention Strategy. Furthermore, this model may be used by other countries, particularly those facing similar public health challenges, offering a pathway to strengthening global suicide prevention efforts.

## Figures and Tables

**Table 1:** Description of the variables included on the national surveillance system for suicide attempts.

Variable	Description
ID number	Random number created to anonymize and protect the identity of the person
Country	Country of origin
Sex	Sex categorized as female or male
Birth date	Birth date (DD/MM/YYYY)
Method of self-harm	Method of self-harm (option list includes hanging or suffocation, handgun discharge, self-poisoning by drugs/medicines, intentional self-harm by sharp object, other); only the main method is recorded, prioritizing the most lethal
Suicide attempt date	Suicide attempt date (DD/MM/YYYY).
Previous suicide attempts	Previous suicide attempts (option list includes yes, no, unknown)
In treatment	Whether the person is already receiving mental health treatment (option list includes yes, no)
Referral to mental health care	Referral to a mental health professional (option list includes yes, no)
Healthcare provider	Patient’s healthcare institution
Registration location	Emergency department where the suicide attempt was recorded
Registration date	Date of registration (DD/MM/YYYY)

## Data Availability

Data handling procedures follow the guidelines established by WHO for sensitive health data management in surveillance systems.
